# Sanguiin H-6 Fractionated from Cloudberry (*Rubus chamaemorus*) Seeds Can Prevent the Methicillin-Resistant *Staphylococcus aureus* Biofilm Development during Wound Infection

**DOI:** 10.3390/antibiotics10121481

**Published:** 2021-12-03

**Authors:** John Jairo Aguilera-Correa, Sara Fernández-López, Iskra Dennisse Cuñas-Figueroa, Sandra Pérez-Rial, Hanna-Leena Alakomi, Liisa Nohynek, Kirsi-Marja Oksman-Caldentey, Juha-Pekka Salminen, Jaime Esteban, Juan Cuadros, Riitta Puupponen-Pimiä, Ramon Perez-Tanoira, Teemu J. Kinnari

**Affiliations:** 1 Clinical Microbiology Department, IIS-Fundación Jiménez Díaz, UAM. Avda. Reyes Católicos 2, 28040 Madrid, Spain; john.aguilera@fjd.es (J.J.A.-C.); jesteban@fjd.es (J.E.); 2 Networking Research Centre on Infectious Diseases (CIBER-ID), 28029 Madrid, Spain; 3 Infectious Diseases and Tropical Medicine Service, IIS-Fundación Jiménez Díaz, UAM. Avda. Reyes Católicos 2, 28040 Madrid, Spain; saraferli.sf@gmail.com (S.F.-L.); iskra.cunnas@estudiante.uam.es (I.D.C.-F.); 4 Neumology Department, IIS-Fundación Jiménez Díaz, Universidad Autónoma de Madrid, CIBERES, 28040 Madrid, Spain; sperezr@fjd.es; 5 VTT Technical Research Centre of Finland Ltd. Tietotie 2, FI-02150 Espoo, Finland; hanna-leena.alakomi@vtt.fi (H.-L.A.); liisa.nohynek@vtt.fi (L.N.); Kirsi-Marja.Oksman@vtt.fi (K.-M.O.-C.); 6 Natural Chemistry Research Group, Department of Chemistry, University of Turku, FI-20014 Turku, Finland; j-p.salminen@utu.fi; 7 Clinical Microbiology Department, Hospital Universitario Príncipe de Asturias, 28805 Madrid, Spain; juan.cuadros@uah.es; 8 Department of Biomedicine and Biotechnology, University of Alcalá, 28805 Alcalá de Henares, Spain; 9 Department of Otorhinolaryngology-Head and Neck Surgery, Helsinki University Hospital, University of Helsinki, P.O. Box 263, 00029 HUS, FI-00130 Helsinki, Finland

**Keywords:** methicillin-resistant *S. aureus*, biofilm, cloudberry, *Rubus*, ellagitannin

## Abstract

*Staphylococcus aureus* is the most common cause of surgical site infections and its treatment is challenging due to the emergence of multi-drug resistant strains such as methicillin-resistant *S. aureus* (MRSA). Natural berry-derived compounds have shown antimicrobial potential, e.g., ellagitannins such as sanguiin H-6 and lambertianin C, the main phenolic compounds in *Rubus* seeds, have shown antimicrobial activity. The aim of this study was to evaluate the effect of sanguiin H-6 and lambertianin C fractionated from cloudberry seeds, on the MRSA growth, and as treatment of a MRSA biofilm development in different growth media in vitro and in vivo by using a murine wound infection model where sanguiin H-6 and lambertianin C were used to prevent the MRSA infection. Sanguiin H-6 and lambertianin C inhibited the in vitro biofilm development and growth of MRSA. Furthermore, sanguiin H-6 showed significant anti-MRSA effect in the in vivo wound model. Our study shows the possible use of sanguiin H-6 as a preventive measure in surgical sites to avoid postoperative infections, whilst lambertianin C showed no anti-MRSA activity.

## 1. Introduction

The recent increase in the number and complexity of surgical interventions increases the number of surgical site infections (SSI) [1,2]. The SSIs are the most common healthcare-associated infections and are associated with prolonged antibiotic therapy, longer post-operative hospital stays, additional surgical procedures, even with treatment in intensive care units and higher mortality [3].

*Staphylococcus aureus* is the most common pathogen causing SSIs [4]. Methicillin-resistant *S. aureus* (MRSA) is isolated in a high percentage of SSIs [4,5,6]. MRSA infections are more severe and require up to five days longer hospitalization compared to those caused by methicillin-sensitive *S. aureus* strains [7], which increases suffering of the patient and higher health care costs [8]. Moreover, SSIs are biofilm-related infections where bacteria are encased in a protective matrix and are becoming more resistant to antimicrobial treatment [9,10].

Microbial resistance is unfortunately increasing and many *S. aureus* strains, among others [11], have gained reduced susceptibility or complete resistance to glycopeptides [12,13]. Therefore, to control the spread of antibiotic resistance and treat microbial infections including SSI it is necessary to invest in research and development of new antimicrobials. The bactericidal non-antibiotic natural products can be found in various sources including plants, insects, animals, and fungi. There are an enormous number of plant extracts that have showed anti-MRSA properties per se. So, for instance, this ability has been recently described in extract of desert thorn (*Lycium shawii*), and Indian gooseberry (*Phyllanthus emblica*), pamp (*Arbutus pavarii*) or mango ginger (*Curcuma amada*) [14,15,16,17].

**Figure 1:**
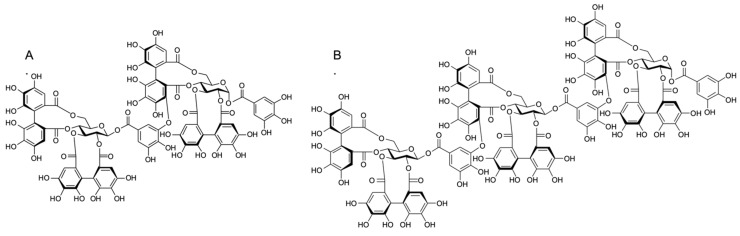
Chemical structure of sanguiin H-6 (**A**) and lambertianin C (**B**).

Natural berries possess phenolic compounds that have shown promising antimicrobial and anti-biofilm effects against MRSA [18]. Among these berry-derived compounds, ellagitannins are particularly interesting. They are molecules that have shown antimicrobial activity against fungi, viruses, and bacteria, including MRSA [19,20]. Ellagitannins are a diverse group of hydrolysable tannins together with gallotannins and simple gallic acid derivatives. They typically contain one or more hexahydroxydiphenoyl (HHDP) groups formed by C-C coupling of two galloyl groups [21]. Further modifications to the HHDP groups and different types of oligomerization reactions between the monomers increase ellagitannin diversity. Sanguiin H-6, an ellagitannin dimer, and lambertianin C, a trimer (Figure 1), are the major ellagitannins present in the extracts of *Rubus* berries such as raspberries (*Rubus idaeus*) and cloudberries (*Rubus chamaemorus*) [22,23]. Ellagitannins are also the main phenolic compounds in *Rubus* seeds. The seeds of cloudberry contain a complex mixture of ellagitannins, including lambertianin C and sanguiin H-6 [24].

The aim of this study was to evaluate the effect of sanguiin H-6 and lambertianin C fractionated from cloudberry seeds, on the MRSA growth, biofilm development in different growth media in vitro and in vivo by using a murine wound infection model using them as a preventive treatment of MRSA wound infections.

## 2. Results

### 2.1. Minimum Inhibitory Concentration and Minimal Bactericidal Concentration

**Figure 2:**
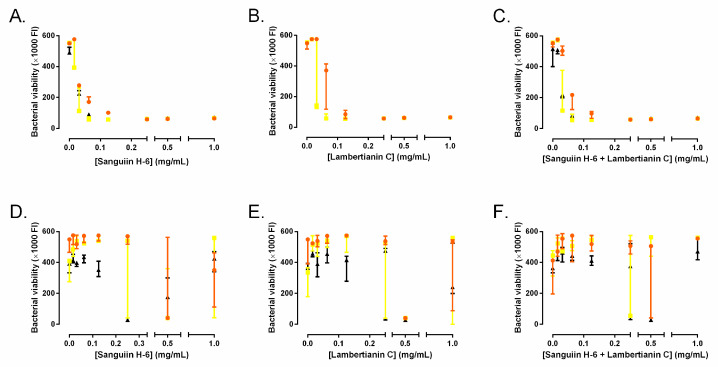
Minimum inhibitory concentrations (**A**–**C**) and minimal bactericidal concentrations (**D**–**F**) of sanguiin H-6 (**A**,**D**) and lambertianin C (**B**,**E**) and their mixture (**C**,**F**) against the three methicillin-resistant *S. aureus* (MRSA) strains: SAP231 (orange circle), MRSA1 (yellow square), and MRSA (black triangle).

MIC of sanguiin H-6 and lambertianin C were 0.25 mg/mL for all three MRSA strains (Figure 2A,B). The MIC of the mixture of sanguiin H-6 and lambertianin C was not significantly different compared to each compound separately (Figure 2C). Therefore, we tested each compound as monotherapy for the following experiments. MBCs of sanguiin H-6 and lambertianin C were greater than 1 mg/mL for all three MRSA strains (Figure 2D–E). MBC of the mixture of sanguiin H-6 and lambertianin C did not show a significant difference (Figure 2F).

### 2.2. Effect of Ellagitannins on the MRSA Growth

**Figure 3:**
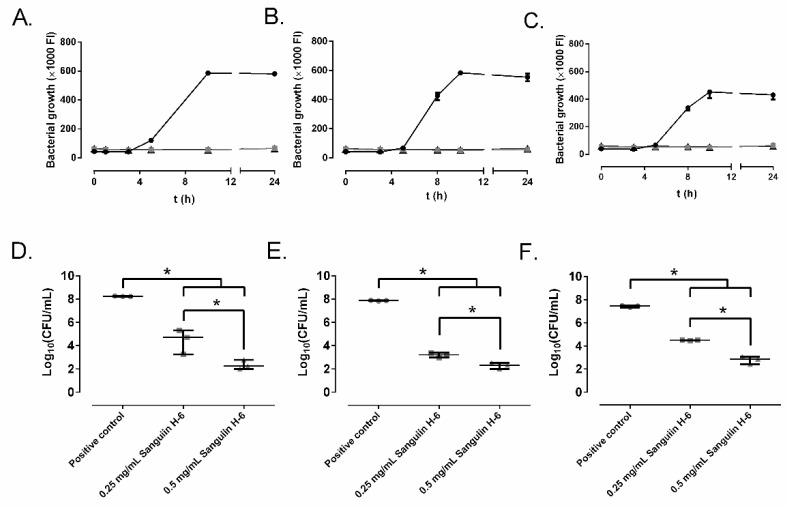
The effect of different concentrations of sanguiin H-6 on SAP231 (**A**,**D**), MRSA 1 (**B**,**E**), and MRSA 2 (**C**,**F**) growth: control (circle), 0.25 mg/mL (square), and 0.5 mg/mL (triangle) over time (**A**–**C**) and at 24 h (**D**–**F**). *: *p*-value < 0.05 for Wilcoxon test.

**Figure 4:**
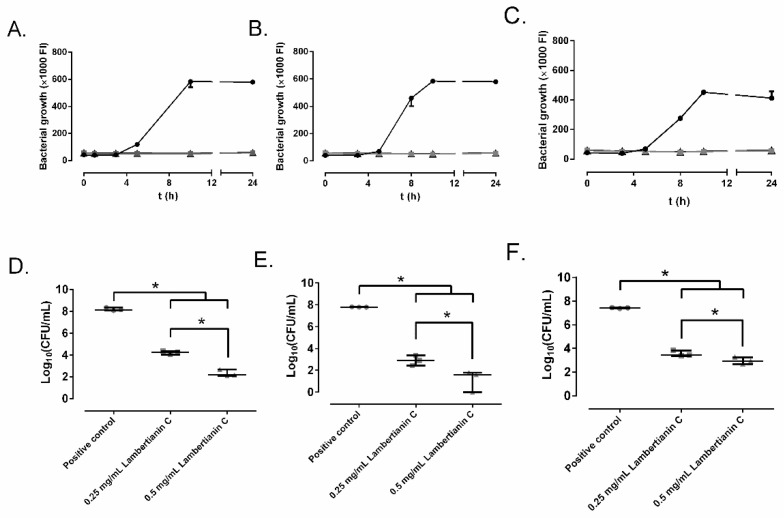
The effect of different concentrations of lambertianin C on SAP231 (**A**,**D**), MRSA 1 (**B**,**E**), and MRSA 2 (**C**,**F**) growth: control (circle), 0.25 mg/mL (square), and 0.5 mg/mL (triangle) over time (**A**–**C**) and at 24 h (**D**–**F**). *: *p*-value < 0.05 for Wilcoxon test.

Figure 3 and Figure 4 represent the bacterial growth curves of sanguiin H-6 and lambertianin C. Both compounds showed a concentration-dependent bactericidal effect and significantly decreased the bacterial concentration of all three MRSA strains. For all MRSA strains, the highest bactericidal concentration of both sanguiin H-6 and lambertianin C was 0.5 mg/mL.

### 2.3. Effect on Biofilm Development

**Figure 5:**
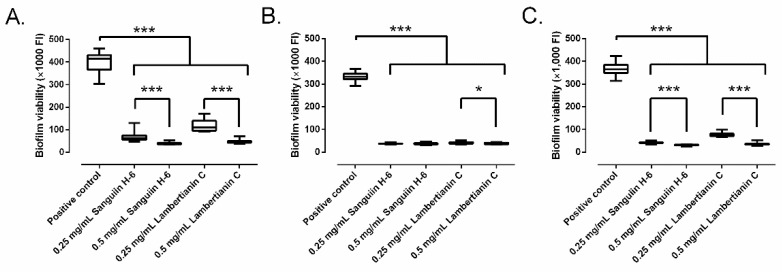
Biofilm development of SAP231 (**A**), MRSA1 (**B**), and MRSA2 (**C**) in presence of sanguiin H-6 and lambertianin C at different concentration:. *: *p*-value < 0.05, ***: *p*-value < 0.001 for Wilcoxon test.

Sanguiin H-6 and lambertianin C showed a significant concentration-dependent inhibitory effect on MRSA biofilm development (Figure 5). Sanguiin H-6 decreased the biofilm growth between 85.1% and 91.1% at 0.25 mg/mL, and between 90.7% and 92.4% at 0.5 mg/mL in all three MRSA strains compared to the control. Interestingly, the inhibitory effect of sanguiin H-6 on MRSA1 was not concentration-dependent (Figure 4B). Lambertianin C decreased the biofilm growth between 73.3% and 90.5% at 0.25 mg/mL, and between 89.1% and 91.5% at 0.5 mg/mL in all three MRSA strains compared to the control.

### 2.4. Biofilm Treatment

**Figure 6:**
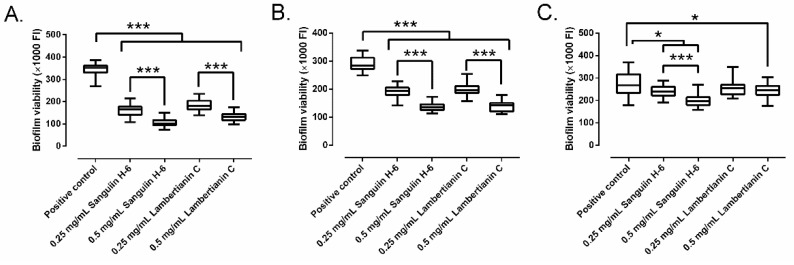
The effect of sanguiin H-6 and lambertianin C at different concentration on growth of a mature 24-h biofilm of SAP231 (**A**), MRSA1 (**B**), and MRSA2 (**C**). *: *p*-value < 0.05, ***: *p*-value < 0.001 for Wilcoxon test.

Sanguiin H-6 and lambertianin C showed a significant concentration-dependent inhibitory effect on the growth of mature 24-h MRSA biofilm (Figure 6). Sanguiin H-6 decreased the biofilm growth between 31.6% and 52.8% at 0.25 mg/mL, and between 44.2% and 71.1% at 0.5 mg/mL compared to the control. Lambertianin C decreased the biofilm growth between 27.3% and 48.4% at 0.25 mg/mL, and between 29.9% and 62.6% at 0.5 mg/mL in all three MRSA strains compared to the control. However, lambertianin C did not inhibit the biofilm growth of MRSA2 significantly at 0.25 mg/mL (Figure 6C).

### 2.5. Biofilm Development in Wound-Like Medium

**Figure 7:**
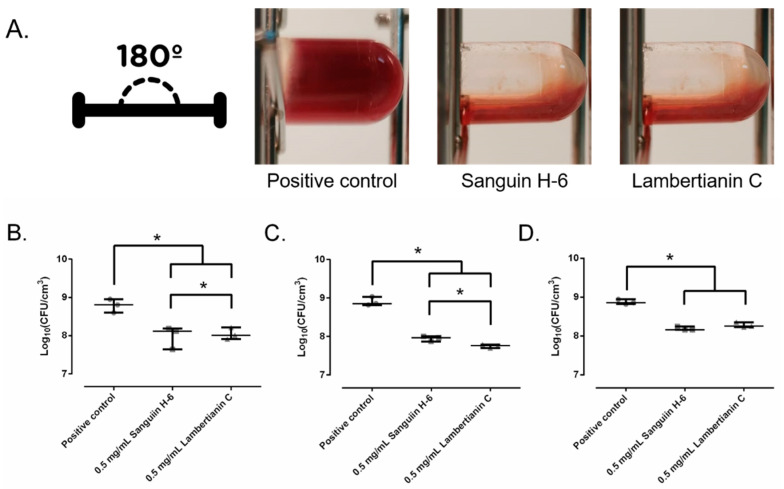
Macroscopic appearance of wound-like medium after the SAP231 biofilm development (**A**). The effect of sanguiin H-6 and lambertianin C at different concentrations on SAP231 (**B**), MRSA1 (**C**), and MRSA2 (**D**) biofilm development in wound-like medium:. *: *p*-value < 0.05 for Wilcoxon test.

In a wound-like medium, sanguiin H-6 and lambertianin C inhibited the coagulation caused by MRSA, as can be seen in Figure 7A. Sanguiin H-6 decreased the biofilm development of the three MRSA strains between 77.2% and 85.3% at 0.5 mg/mL compared to the control while lambertianin decreased it between 71.9% and 90.9% at 0.5 mg/mL, respectively. Supported by these results, the in vivo model was based on MRSA infection prevention.

### 2.6. In Vivo Studies

**Figure 8:**
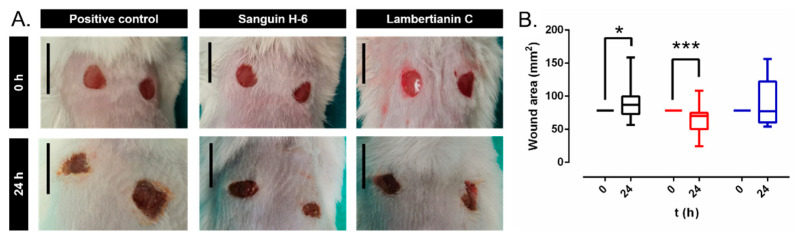
(**A**) Visual appearance of infected wound. It is noteworthy that the difference of the area between wounds of the same mouse is a result of each animal tending to focus on and compulsively scratch one wound more than another. (**B**) The wound area measurements in each experimental group: positive control (black), sanguiin H-6 (red), and lambertianin C (blue). *: *p*-value < 0.05, ***: *p*-value < 0.001 for paired Wilcoxon test.

Figure 8A shows the appearance of the mice wound. Thereafter 24 h, the wound area increased significantly 11% in the control group (*p*-value = 0.0206) and decreased significantly 10.5% in the sanguiin H-6 treated group (*p*-value = 0.0206) (Figure 8B). Lambertianin C did not alter wound area (*p*-value = 1.0000) (Figure 8B).

**Figure 9:**
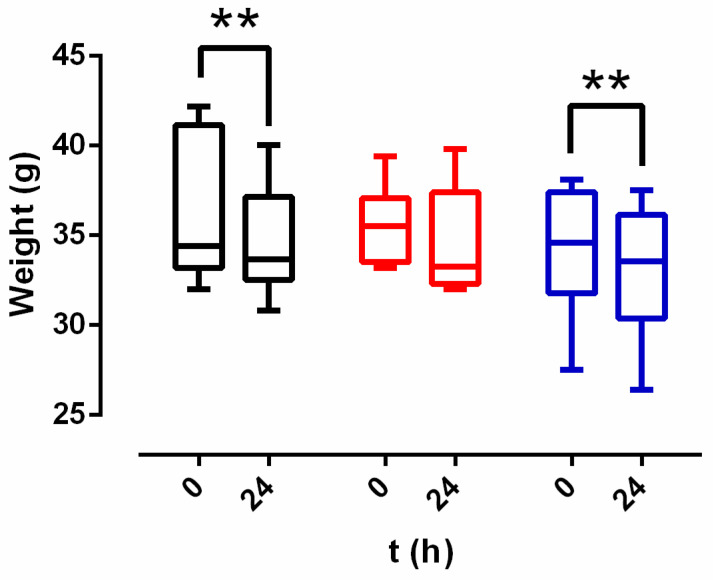
Weight results of each experimental group: positive control (black), sanguiin H-6 (red), and lambertianin C (blue). **: *p*-value < 0.01 for paired Wilcoxon test.

Mice from control and lambertianin C groups lost weight after 24 h (*p*-value = 0.0011 for both of groups) (Figure 9). This weight loss was 1.3 (0.6–1.5) g for the control group and 1.15 (0.5–2.15) g for lambertianin C group. There was no weight alteration in the sanguiin H-6 group (*p*-value = 0.1527) (Figure 9).

**Figure 10:**
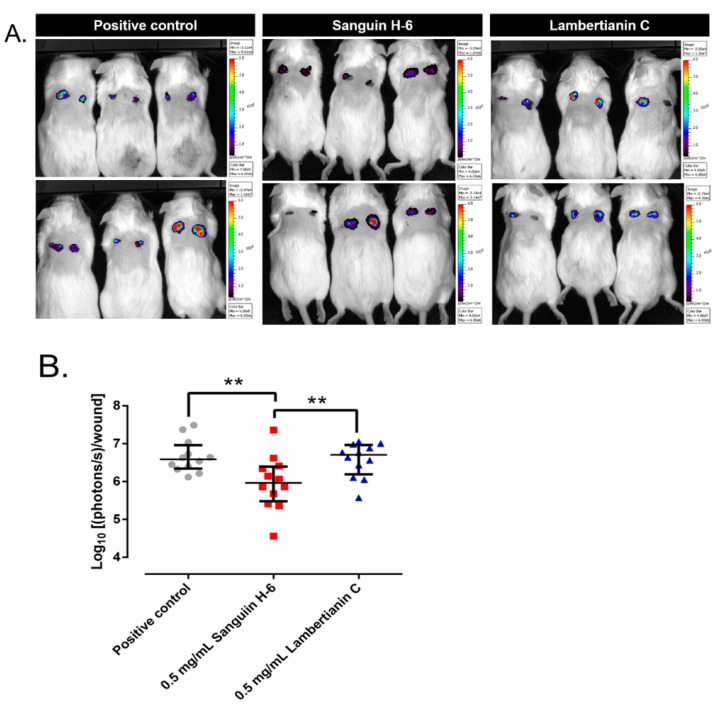
(**A**) Bioluminescence emitted by SAP231 (photons/s/cm^2^) in the infected wounds of each group of mice. It is noteworthy that the bioluminescence from each wound is directly proportional to the number of live staphylococci. (**B**) Wound area in each experimental group. **: *p*-value < 0.01 for Wilcoxon test.

The bioluminescent images appear in Figure 10A. The numerical results of the luminescent emission analysis are represented in Figure 10B. Sanguiin H-6 significantly decreased the bioluminescence 10% with respect to the positive control group (*p*-value = 0.0023) and 11.1% with respect to the lambertianin C group (*p*-value = 0.0086). Lambertianin C increased the bioluminiscence slightly but significantly 1.8% with respect to the positive control group (*p*-value = 0.0086).

## 3. Discussion

In this study, we report the ability of sanguiin H-6 and lambertianin C, two oligomeric ellagitannins, to prevent the biofilm development of methicillin-resistant *Staphylococcus aureus* (MRSA). This could be verified using two different in vitro methods: biofilm development in tryptic-soy broth supplemented with 0.5% glucose and in wound-like medium. Moreover, we report the ability of sanguiin H-6 to decrease the wound infection development of MRSA in vivo.

The MIC was 0.25 mg/L for both sanguiin H-6 and lambertianin C, whilst MBC was greater than 1 mg/mL for sanguiin H-6 and 0.5 mg/mL for lambertianin C. The finding considering sanguiin H-6 is consistent with results reported previously [20,25,26], however, we have not found earlier published studies on MIC and MBC for Lambertianin C against MRSA.

The anti-MRSA effect of sanguiin H-6 and lambertianin C seems to be due to a concentration-dependent bactericidal mechanism since both at 0.5 mg/mL showed reduction higher than 99.9% (Figure 2 and Figure 3) for the three MRSA tested [27]. Sanguiin H-6 is a potent inhibitor of DNA topoisomerase II-mediated DNA unknotting while it binds to topoisomerase II, non-covalent topoisomerase II-DNA intermediate, or both [28]. Curiously, the eukaryotic and prokaryotic type IIA topoisomerases (DNA-gyrase for *S. aureus*) conserve the residues at the DNA cleavage catalytic center [29]. Therefore, we hypothesize that MRSA DNA-gyrase inactivation produces an anti-MRSA effect of sanguiin H-6 with an antibacterial mechanism analogue to other compounds such as etoposide or aminocoumarin. Etoposide is a semi-synthetic podophyllin derived from the mayapple (*Podophyllum peltatum* L.) which can inhibit the DNA gyrase of tumor cells and bacteria [30,31]. The crystal structures show that the way etoposide binds to human topoisomerase II and *S. aureus* gyrase is very similar [29]. Aminocoumarins are structurally related to antibiotics produced by different *Streptomyces* strains, which are potent inhibitors of bacterial gyrase and their binding sites and mode of action differ from those of fluoroquinolones [32].

Bacterial biofilm formation in SSI begins when planktonic bacteria find their way to colonize the surgical site. Once there, they attach themselves to exposed subcutaneous tissue thanks to different microbial surface components recognizing adhesive matrix molecules present in cell walls. After the attachment, the microcolony growth and biofilm formation starts including the synthesis of a protective glycocalyx matrix that encases the colony. Our biofilm development study showed that sanguiin H-6 and lambertianin C were able to inhibit completely the MRSA biofilm development and although less significantly, the highest concentrations of sanguiin H-6 and lambertianin C were able to decrease the MRSA biofilm growth.

The wound-like medium provides a more realistic in vitro biofilm model simulating some of the functional characteristics of chronic pathogenic biofilms and allows the development of new tools for fighting against these infections [33]. Interestingly, the presence of sanguiin H-6 and lambertianin C prevented the coagulation of the wound-like medium. This may be due to at least two reasons. Firstly, ellagitannins can interact with plasminogen and avoid its cleavage from plasminogen to plasmin carried out by staphylococcal coagulases [34], as well as gallic acid and other simple phenolics which can interact with thrombin [35]. Secondly, sanguiin H-6 and lambertianin C may exert slightly bactericidal or bacteriostatic effects in the wound-like medium that would decrease the coagulase’s production. The biofilm matrix protects the bacteria [9], however, the highest concentrations of sanguiin H-6 and lambertianin C were able to significantly decrease the biofilm development in the wound-like medium as well.

In the in vivo study, sanguiin H-6 demonstrated a light but significant anti-MRSA effect, something reflected in the three aspects evaluated: wound area, weight loss and bacterial amount per wound. Firstly, the wound area decreased in the sanguiin H-6 group and increased in the control group. Secondly, the sanguiin H-6 group was the only one which maintained weight after infection. The time spent in sleeping or scratching the infected wound during the day, and reduced appetite and consumption of food and water produced the weight loss of other groups [36]. Thirdly, the sanguiin H-6 group showed the lowest bacterial amount per wound. Interestingly, the reduction in the wound area and the MRSA amount per wound were proportional (10.5% and 10%, respectively) in the sanguiin H-6 group, which leads us to reason that the apparent wound area reduction is directly related to the anti-MRSA effect of sanguiin H-6 and not to a potential anti-inflammatory or other healing effect. The results of the in vivo experiments are straightforward in showing their anti-MRSA activities. However, lambertianin C showed no anti-MRSA activity, so it could not be used as an antiseptic or topical agent against MRSA wound infections. On the contrary, sanguiin H-6 can be used as an antiseptic or a topical agent rather than an antibiotic or as an adjuvant in conjunction with an anti-MRSA antibiotic (e.g., mupirocin) or antiseptic (e.g., chlorhexidine).

This study shows at least one limitation since chlorhexidine or mupirocin should have been used as a therapeutic control in the in vivo model. Recently, some plant extracts have shown a synergetic anti-staphylococcal effect in combination with some antibiotics, e.g., saponin fraction of *Tetraena alba* and penicillin and isoflavonoids of hairy-fruited broom (*Cytisus striatus*) and ciprofloxacin or erythromycin [15,37,38].

## 4. Materials and Methods

### 4.1. In Vitro Studies

#### 4.1.1. Ellagitannins Extraction

Cloudberry material. Dry cloudberry (*Rubus chamaemorus*) press cake material originated from commercial juice pressing processes of Finnish origin, and it was obtained from Kiantama company (Kiantama Oy, Suomussalmi, Finland), specialized in wild berry processing. The dry press cake was stored at +15 °C until further processed.

Seed sanding. A dried pressed cake of cloudberry juice pressing process was firstly sieved as a pre-treatment for sanding. The seeds were separated from the skin fraction by using a vibratory sieve shaker (Retsch AS 200 control, Retsch GmbH, Haan, Germany) with a 1.6 mm screen at settings of 5–10 min sieving time and 1.5 mm amplitude. An abrasive machine (barley pearling machine, tailor-made for VTT) sanded the seeds for 15 min resulting in a light-yellow seed coatpowder, which was used in further experiments, the peeled seeds remaining intact and whole after the sanding process, and suitable for further processing. The detailed sanding process is described by Puupponen-Pimiä et al. [20,39].

Extraction and purification. An amount of 300 g of seed coat powder was extracted six times with 4.8 L of acetone/water (80:20, *v*:*v*), reduced to water phase, filtered and freeze-dried. Freeze-dried extract (20 g) was purified in 1 g aliquots with preparative liquid chromatography as described e.g., in Baert et al. [25]. We analyzed the collected fractions by UHPLC-MS/MS to study their ellagitannin composition. Fractions with similar composition were pooled to finally obtain seven fractions, including purified sanguiin H-6 and lambertianin C, and their mixture.

#### 4.1.2. Bacteria

We studied three different MRSA strains related to SSI. A genetically modified wild-type USA300 strain (SAP231), for use in bioluminescent imaging, and two clinical stains isolated in the microbiology department of the Fundación Jiménez Díaz University Hospital [40]: MRSA 1 isolated from a 73-year-old man with an infected wound, and MRSA 2, isolated from paronychia of a 92-year-old man. All strains were stored frozen at −80 °C until experiments were performed.

#### 4.1.3. Minimum Inhibitory Concentration and Minimum Bactericidal Concentration

Minimum inhibitory concentrations (MIC) were determined using previously described broth microdilution method with some modifications [27]. In brief, a series of two-fold dilutions of ellagitannins in concentration from 1 mg/mL to 15.625 µg/mL were added to cation-adjusted Müeller-Hinton broth (Sigma Aldrich, St. Louis, MO, USA) to a final volume of 100 μL per well to a Costar 96-well round-bottom polypropylene plate (Corning Inc., Corning, NY, USA). Thereafter, 100 µL of bacterial suspension in cation-adjusted Müeller-Hinton broth (CAMHB) containing approximately 1.6 × 10^6^ colony-forming units per milliliter (CFU/mL) were added followed by static incubation at 37 °C and 5% CO_2_. After incubation, MIC was determined using a modification of the methodology previously described by other authors [41]. For that, 100 μL from each well was mixed with 100 μL of CAMHB with 20% alamarBlue (BIO-RAD, Hercules, CA, USA) in a new 96-well plate reaching a final concentration of 10% of alamarBlue and incubated at 37 °C and 90 rpm for 30 min [42,43]. On completion of the incubation, columns with no color change were scored, as above the MIC value and the fluorescence was measured using excitation wavelength of 560 nm and emission wavelength of 590 nm.

Minimum bactericidal concentration (MBC) was determined using previously described broth microdilution method with some modifications [44]. Briefly, 20 μL of each well after 24-h incubation were mixed with 180 μL of tryptic soy broth in a new 96-well plate and incubated statically at 37 °C and 5% CO_2_ for 24 h. After incubation, 100 μL of each well were mixed with 100 μL of tryptic soy broth with 20% alamarBlue, added to a new 96-well flat-bottom plate and incubated at 37 °C and 90 rpm for 30 min. Thereafter, the fluorescence was measured using excitation wavelength of 560 nm and emission wavelength of 590 nm. These assays were performed in triplicate. MBC results were also evaluated by plating a drop of 10 µL in a mannitol-salt agar plate (Biomérieux, Îlle de France, France) after reading MIC using alamarBlue.

#### 4.1.4. Effect of Ellagitannins on the MRSA Growth

Of each MRSA strain, 200 µL were incubated with 4 mL of CAMHB (10^6^ CFU/mL) with or without ellagitannin (positive control) at 37 °C and 5% CO_2_ for 24 h in a 12-well polypropylene plate (Thermo Fisher Scientific, Waltham, MA, USA). The bacterial viability was periodically measured at 1, 3, 6, 12, and 24 h by taking a 100 µL sample from each well and mixing it with 100 µL of CAMHB supplemented with 20% of alamarBlue in a Nunc™ 96-well flat-bottom polypropylene MicroWell™ plate. The plate was incubated at 37 °C and 90 rpm for 30 min and the fluorescence was measured thereafter-using excitation wavelength of 560 nm and emission wavelength of 590 nm. After the last measurement at 24 h, the number of CFU per milliliter was estimated using the drop plate method in mannitol-salt agar plates. This experiment was performed in triplicate for each strain (*n* = 3).

#### 4.1.5. Effect on Biofilm Development

An overnight culture of each strain was grown in tryptic soy agar at 37 °C and 5% CO_2_. Thereafter it was resuspended in saline to 10^6^ CFU/mL and 100 μL was incubated at 37 °C and 5% CO_2_ for 90 min in static conditions in 96-well flat-bottom plate. After the incubation, each well was rinsed two times with 100 μL of saline. Thereafter, in each well 150 μL of tryptic soy broth with 0.5% glucose as biofilm inductive growth medium was added with or without ellagitannin (positive control) at different concentrations and incubated at 37 °C and 5% CO_2_ for 24 h [45]. After incubation, each well was rinsed two times with 100 μL of saline and thereafter 150 μL of tryptic soy broth with 10% alamarBlue was added and incubated at 37 °C and 90 rpm for 30 min. After incubation, the fluorescence was measured using an excitation wavelength of 560 nm and emission wavelength of 590 nm. This experiment was performed in eight wells per ellagitannin concentration and in triplicate for each strain (*n* = 24). The most effective concentration was used in the following experiments.

#### 4.1.6. Biofilm Treatment

For developing a mature biofilm, 100 µL of each strain, resuspended in saline to 10^6^ CFU/mL, was incubated at 37 °C and 5% CO_2_ for 90 min in static conditions in a 96-well flat-bottom plate. After incubation, each well was rinsed two times with 100 μL of saline, and thereafter 150 μL of tryptic soy broth with 0.5% glucose was added and the plate was incubated at 37 °C and 5% CO_2_ for 24 h. After 24-h incubation, each well was rinsed two times with 150 μL of saline. Then, 150 μL of tryptic soy broth with 0.5% glucose, with or without (positive control) each ellagitannin at different concentrations, was incubated at 37 °C and 5% CO_2_ for 24 h. After incubation, each well was rinsed two times with 100 μL of saline and thereafter 150 μL of tryptic soy broth with 10% alamarBlue was added and incubated at 37 °C and 90 rpm for 30 min. After incubation, the fluorescence was measured using an excitation wavelength of 560 nm and emission wavelength of 590 nm. This experiment was performed in eight wells per ellagitannin concentration and by triplicate for each strain (*n* = 24). The most effective concentration was used in the following experiments.

#### 4.1.7. Effect on Biofilm Development in Wound-like Medium

Biofilm development in wound-like medium was determined using previously described methods based on the Lubbock chronic wound medium [33,46]. Wound-like medium was composed of 45% Bolton broth (Sigma-Aldrich, St. Louis, MO, USA), 50% bovine plasma (Sigma-Aldrich), 5% lacquered horse red blood cells (Thermo Fisher Scientific), supplemented with one lyophilized of BD BBL™ coagulase plasma, and with or without (positive control) each ellagitannin in different concentrations. One milliliter of each medium was incubated with 50 µL of 10^8^ CFU/mL of each MRSA strain in saline at 37 °C and 5% CO_2_ for 24 h in 12-well plates. After incubation, the content of each well was sonicated in a 50-mL Costar™ conical tube (Corning Inc.) with 10 mL of saline, with an Ultrasons-H 3,000,840 low-power bath sonicator (J. P. Selecta, Abrera, Spain) at 22 °C for 5 min. This sonicated saline was serially diluted with saline and CFU per milliliter was estimated using the drop plate method in mannitol-salt agar plates. This experiment was performed in triplicate for each strain (*n* = 3).

### 4.2. In Vivo Studies

This study was approved by the Fundación Jiménez Díaz-University Hospital Health Research Institutes Committee of Animal ethics and welfare, which includes ad hoc members for ethical issues. Animal care and maintenance complied with institutional guidelines as defined in national and international laws and policies (Spanish Royal Decree 53/2013, authorization reference PROEX 145/18 21 September 2018, granted by the Counsel for the Environment, Local Administration and Territorial Planning of the Community of Madrid and, Directive 2010/63/EU of the European Parliament and of the Council of 22 September 2010).

**Figure 11:**
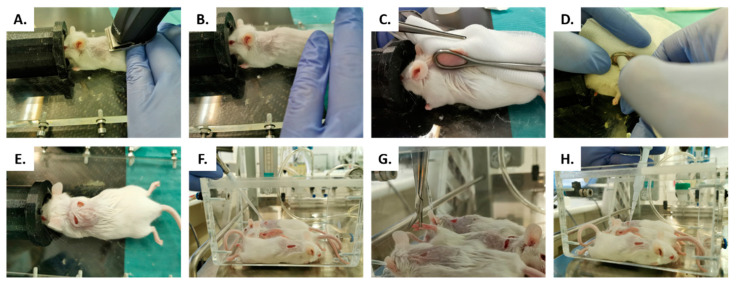
In vivo wound model, the interscapular skin was completely shaved (**A**) and disinfected with 70% ethanol (**B**). Two 5-mm full-thickness excisional wounds were created using a non-toothed Foerster caliper (**C**) and a 5-mm biopsy punch (**D**,**E**). The wounds of untreated group were compared to groups treated with: Sanguiin H-6 or lambertianin C (**F**). During this preventive treatment, we cut the nails of hind legs (**G**). After treatment, each wound was infected with SAP231 MRSA strain (**H**).

Surgical model was inspired by the wound infection model previously described by Wang et al. [47]. Briefly, 12-week-old male SWISS RjOrl: SWISS (CD1^®^) mice (Janvier Labs, Le Genest-Saint-Isle, France) were anesthetized with 2.5–3.5% of isoflurane. Then, the interscapular skin was completely shaved and disinfected with 70% ethanol (Figure 11A,B). Two 5-mm full-thickness excisional wounds were created on the back of each mouse on each side of the spine, using a non-toothed Foerster caliper and a 5-mm biopsy punch (GSK, London, United Kingdom) (Figure 2C–E). Mice were distributed into three groups depending on the preventive wound treatment: The wounds were treated for 15 min either with saline (positive control, *n* = 6) or with 20 μL of the two ellagitannins, Sanguiin H-6 (*n* = 6) or Lambertianin C (*n* = 6) both in concentration of 0.5 mg/mL (Figure 11F). During this preventive treatment, nails of the hind legs were cut (Figure 2G). Thereafter, each wound was infected with 2 μL of the luminescent SAP231 strain suspension in concentration of 3.4 × 10^8^ CFU/mL in saline for 15 min (Figure 2G). After this time, the mice were placed in a sterile cage with cellulose bedding and an additional mixture of grains and vegetables (Vitakraft, Bremen, Germany).

The weight and wound ellipsoidal area were measured at 0 h and at 24 h. After 24 h, mice from all groups were imaged using the IVIS-Lumina Imaging System (PerkinElmer, Waltham, MA, USA). Total photon emission from predefined regions of interest (ROI) of the wound was acquired using the maximum exposition time recommended by the software, which was always less than 5 min. Captured emission was then quantified as photons per second per wound by using the Living Image software package (Xenogen Corp., Alameda, CA, USA) [48]. Bioluminescence was expressed in the images using a pseudocolor scale, with red representing the most intense luminescence and blue representing the least intense luminescence; the data are presented as cumulative photon counts collected from each ROI [48]. After the final imaging time point, the animals were euthanized by hypercapnia.

#### Statistical Analysis

Statistical analyses were performed using Stata Statistical Software, Release 11 (2009) (StataCorp, College Station, TX, USA). All data were evaluated using a one-sided unpaired Wilcoxon nonparametric test to compare two groups, except to in vivo model data of weight and wound area which was evaluated using a one-sided paired Wilcoxon nonparametric test. Statistical significance was set at *p*-values ≤ 0.05. Body weight was evaluated over time using a linear regression model. The values are cited and represented as median and interquartile range.

## 5. Conclusions

In conclusion, the biofilm development and growth of MRSA was significantly inhibited by sanguiin H-6 and lambertianin C in vitro studies. Furthermore, sanguiin H-6 showed significant anti-MRSA effect in the in vivo wound model. Our study shows that oligomeric ellagitannins such as sanguiin H-6 where monomers are linked by galloyl to hexahydroxydiphenoyl (HHDP) linkage (so called GOD linkage) could be used as a preventive measure in surgical sites to avoid postoperative infections. More studies will be needed to elucidate the real molecular mechanism of sanguiin H-6 inhibition.

## 6. Patents

Puupponen-Pimiä, R.; Kössö, T.; Nohynek, L.; Mokkila, M. Process for converting berry and fruit material to antimicrobially active fractions. WO2016/097488.

## Data Availability

Data is contained within the article.
